# Can spa rehabilitative interventions play a role for patients suffering from neurodegenerative disorders at the early stages? A scoping review

**DOI:** 10.1007/s00484-022-02369-0

**Published:** 2022-09-21

**Authors:** Maria Chiara Maccarone, Stefano Masiero

**Affiliations:** 1 Physical Medicine and Rehabilitation School, University of Padova, Padua, Italy; 2 Rehabilitation Unit, Department of Neuroscience, University of Padova, Padua, Italy

**Keywords:** Thermal water, Aquatic exercise, Parkinson’s disease, Thermal environment, Early rehabilitation, Cost-effectiveness

## Abstract

The global burden of neurodegenerative disorders is significantly increasing as life expectancy rises but currently there is no cure for these conditions. An extensive search on MEDLINE (PubMed) and PEDro databases was conducted selecting clinical trials, Randomized Controlled Trials, and longitudinal studies published in the last 20 years in order to highlight what evidence there is for a role of spa rehabilitative interventions for patients with neurodegenerative diseases, in terms of motor function, symptoms, and quality of life (QoL) improvement and cost-effectiveness. A total of 225 publications were analyzed. Only three manuscripts were selected for review because they matched the inclusion criteria. These studies demonstrated statistically significant differences in the outcomes evaluated among patients affected by Parkinson’s disease after thermal rehabilitative treatments: motor function, balance, QoL, and psychological well-being statistically improved. In addition, rehabilitation in the spa setting seemed to be cost-effective for these patients. However, further studies are needed to define the role of spa rehabilitative interventions for these patients as the literature is still limited.

## Introduction

The global burden of neurodegenerative disorders, such as Alzheimer’s disease, Parkinson’s disease (PD), and amyotrophic lateral sclerosis, is increasing significantly as life expectancy rises (Cova et al. 2017). For example, from 1990 to recent years, PD prevalence and disability-adjusted life-years (DALYs) rates increased globally except for a few regions (Dorsey et al. 2018) with a total economic burden in the USA alone of $25.4 billion for the direct medical costs and of $26.5 billion for indirect and non-medical costs (W. Yang et al. 2020).

Despite they affect millions of people worldwide with an increasing trend (Cova et al. 2017; Dorsey et al. 2018; Fereshtehnejad et al. 2019), currently there is no cure for these conditions that are characterized by irreversible and progressive loss of neuronal cells in brain, resulting in cognitive impairment and motoneuron dysfunction (Jellinger 2010). Therefore, an important role is played by interventions aimed at counteracting limitations and preventing disabilities, starting from the early stages.

Exercising has been shown to be beneficial in several neurodegenerative diseases. For example, in PD, one of the most frequent neurodegenerative disorders with still no treatment avoiding disease progression, evidence suggests that exercise can help both motor and nonmotor symptoms (Xu et al. 2019). To enhance gait spatiotemporal parameters including velocity and step amplitude, numerous land-based physiotherapy procedures have been developed and employed (Vivas et al. 2011). However, in these patients, fear of falling as a result of postural instability and movement impairment represents a significant barrier to participation in exercise programs (Plecash & Leavitt, 2014). Therefore, since water is a unique medium that allows mobility and stability exercise with a decreased risk of falling, water-based exercise protocols could be useful for neurodegenerative disorders. The aquatic setting could be further advantageous for patients with neurodegenerative disorders because it provides adequate resistance and encourages strengthening while contributing to minimize harm (Masiero et al. 2020a, b). In particular, in recent years, water-based rehabilitation strategies are increasingly being used for patients with PD and they seem to contribute to improve functional mobility, quality of life (QoL) and motor symptoms such as freezing of gate and balance impairment in these subjects (Ayán & Cancela 2012; Clerici et al. 2019; Palamara et al. 2017).

Aquatic rehabilitative interventions have been demonstrated to be safe and associated with significant gains on motor function and symptomatic relief of spasticity also in patients with multiple sclerosis (de Sa et al. 2011; Plecash & Leavitt 2014). Due to the observed reduction in choreic motions, gentle exercises in warm water were also recommended in patients with Huntington’s disease (Plecash & Leavitt 2014).

Although rehabilitation treatments in thermal water have traditionally been aimed at patients with orthopedic disabilities, recently there has been an increase in real-life spa rehabilitative applications in the neurological field. Exploiting the synergies between the physical effect of immersion (mostly related to temperature, buoyancy, viscosity, and hydrostatic pressure), the benefits of exercise, and the peculiar chemical effects of thermal waters (Fioravanti et al. 2017; Maccarone et al. 2021a, b), several protocols for neurodegenerative disorders rehabilitation in thermal environments are being developed. The aim of this scoping review is to preliminarily investigate this topic, searching the literature for evidence supporting the real-life applications of spa rehabilitative interventions on patients with neurodegenerative disorders, especially in terms of motor function, symptoms, and QoL improvement and cost-effectiveness.

## Materials and methods

### Search strategy

**Fig. 1:**
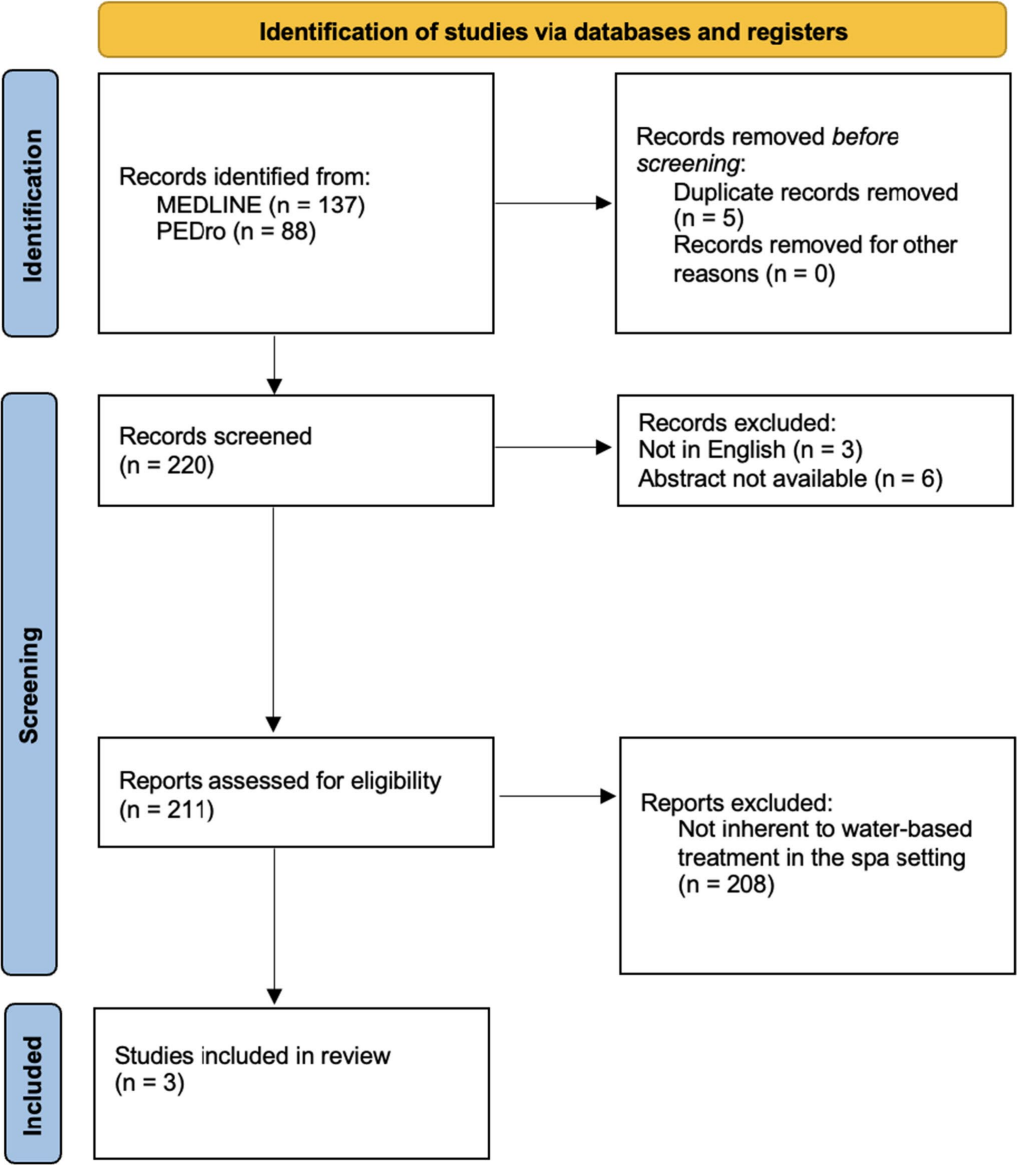
PRISMA 2020 flow diagram for new reviews which included searches of databases (Page et al. 2021)

A scoping review was conducted aimed at investigating evidence for a role of spa rehabilitative interventions among patients with neurodegenerative disorders, in particular at the early stages. The first author established the search question in collaboration with the other author who have clinical and research experience on the topic. The search question was defined as “what evidence is there of a role for spa rehabilitative interventions among patients with neurodegenerative disorders?” PEDro (therapy: hydrotherapy, balneotherapy; subdiscipline: neurology; method: clinical trial) and MEDLINE (Medical Subject Headings—MeSH terms combined with the Boolean operator AND) databases were employed for the search (Table 1). An initial search revealed that trials conducted in the spa setting included only PD patients. As a result, it was agreed to further investigate employing MEDLINE Mesh Terms that explicitly considered PD. Furthermore, because the topic is innovative, an additional search in the grey literature was conducted in order to identify as many studies as possible relating to the topic under consideration. Once the research question had been defined, a thorough procedure of identifying and choosing appropriate studies was carried out (Fig. 1).

**Table 1:** Search strings used to screen clinical trials on MEDLINE

Search strings
(balneotherapy [MeSH Terms]) AND (neurodegenerative disease [MeSH Terms])
(spa therapy [MeSH Terms]) AND (neurodegenerative disease [MeSH Terms])
(thermal environment [MeSH Terms]) AND (neurodegenerative disease [MeSH Terms])
(balneotherapy [MeSH Terms]) AND (Parkinson’s disease [MeSH Terms])
(spa therapy [MeSH Terms]) AND (Parkinson’s disease [MeSH Terms])
(thermal environment [MeSH Terms]) AND (Parkinson’s disease [MeSH Terms])

*PDQ-39*, Parkinson’s Disease Quality of Life Questionnaire; *SF-36*, Medical Outcomes Study 36-item Short Form; *GHQ-28*, General Health Questionnaire; *UPDRS*, Unified Parkinson’s Disease Rating Scale; *BBS*, Berg Balance Scale; *PDQ8*, Parkinson’s Disease Questionaire-8; *Tinetti*, Tinetti balance assessment tool Scale; *BESTest*, Balance Evaluation Systems Test; *ADL*, Activities of Daily Living; *IADL*, Instrumental Activities of Daily Living; *PD-CFRS*, Parkinson’s Disease Cognitive Functional Rating Scale; *NFOG-Q*, New Freezing of Gait Questionnaire; *QoL*, quality of life; *MDS-UPDRS part III*, Unified Parkinson’s Disease Rating Scale part III; *Mini-BESTest*, Mini Balance Evaluation System Test; *TUG*, Timed Up and Go; *DT*, dual-task paradigms; *6MWD*, six minutes walk distance

Original research articles published from January 1, 2002, to March 1, 2022, were included in the review.

### Study selection

The PICO framework was used to answer the research question (P – patient, problem or population: subjects with neurodegenerative disorders, I – Intervention: rehabilitative interventions in the spa setting, C – Comparison, control or comparator: not needed, O – Outcomes: clinical outcomes (motor function modifications, symptom relief, changes in QoL) and cost-effectiveness. Human-based clinical trials, Randomized Controlled Trials (RCTs), and longitudinal studies were considered. All the studies selected for the review were required to have an available abstract. Articles published in languages other than English were not considered.

Titles and abstracts of all the studies found were examined for relevance to the research topic. Studies that did not deal with interventions in thermal water but consider warm water interventions were excluded, as were all studies that did not consider water-based interventions or neurodegenerative disorders.

Following the completion of the study selection, the authors downloaded the full-text version of the articles and screened them individually. Duplicates as well as articles that did not meet the inclusion criteria were eliminated.

### Data extraction

Data were extracted and graphed. Authors, year of publication, neurodegenerative disorders considered, stage of the disease, sample size, intervention under study, and significance of the results were all extracted and gathered in a table (Table 2).

**Table 2:** Main data from the included studies

Authors	Year	Disease	Stage	Population	Intervention	Results
Brefel-Courbon C et al.	2003	PD	Hoehn and Yahr 2: 25 patients (80.64%)	31 patients	A 20-week spa period, including spa therapy for 3 weeks employing water rich in sulfates, calcium and magnesium, followed by a 20-week non-spa period versus the reverse	After 4 weeks: PDQ-39 (stigma *p* = 0.01 and communication *p* = 0.02), SF-36 physical and mental dimension (*p* = 0.03 and *p* = 0.001), part IV of the UPDRS (*p* = 0.03), and GHQ-28 (*p* = 0.004) statistically improved After 20 weeks: no differences Mean direct medical cost over 20 weeks (euro: 1328 ± 167; pound 776 ± 97 per patient) in the spa period was slightly but significantly reduced in comparison with that of the non-spa period (euro: 1380 ± 523; pound 807 ± 306 per patient)
Masiero S. et al.	2019	PD	Hoehn and Yahr 1.5 ± 0.5	14 patients	4 weeks of functional re-education and kinesitherapy in a salso-bromo-iodic pool	At the end of the training UPDRS (*p* = 0.0005), BBS (*p* = 0.0078), PDQ8 (*p* = 0.0039), Tinetti (*p* = 0.0068), and Mini BESTest (*p* = 0.0002) statistically improved
Di Marco et al.	2022	PD	Hoehn and Yahr 2 [2–3]	14 patients	12 sessions lasting 45 min of thermal water exercises, twice a week for 6 weeks, in a 1.4 m depth pool at 32–36 °C	At the end of the treatment, ADL, IADL and PD-CFRS, and NFOG-Q reported no statistically significant differences PDQ-39 score detected significant changes after therapy in the participants’ QoL, mobility and ADL (*p*-values equal to 0.029, 0.040, and 0.049, respectively) The MDS-UPDRS part III score (*p* = 0.022), the Mini-BESTest total score (*p* = 0.0002) significantly improved The TUG test highlighted significant differences in the time to complete the task in both single and dual-task conditions (*pTUG* = 0.002 and *pTUG* − *DT* < 0.001) and TUG timing was significantly reduced (*p* = 0.002) The 6MWD augmented by 51 m (*p* = 0.025)

## Results

A total of 225 publications was found in the preliminary screening process. Of these, only three manuscripts were considered for review because they met the inclusion criteria (Fig. 1).

The selected articles differed in type of rehabilitation treatments examined, frequency, and duration of interventions (Table 2). Moreover, timing, type of assessments, and study design were different between the two trials.

Brefel-Courbon et al. considered PD patients who underwent a spa rehabilitative program, involving thermal baths, drinking mineral water, various types of showers, and underwater massages every morning for 6 days a week. Spa therapy was conducted in a spa resort of Ussat Les Bains (Ariege area, southwest of France); the water employed was rich in sulfates, calcium, and magnesium. The patients, mainly at the early stages of PD according to the Hoehn-Yahr score, underwent either a spa period (spa therapy for 3 weeks and usual routine drug therapy for 17 weeks) followed by a non-spa period (usual drug therapy only for 20 weeks) or the reverse (delayed spa therapy group), in a randomized, single-blind, cross-over trial. The primary outcome was QoL evaluated through the Parkinson’s Disease Questionnaire (PDQ-39) and the 36-item Short Form Health Survey (SF-36). Secondary outcomes were motor (Unified Parkinson’s Disease Rating Scale (UPDRS)) and psychological function (General Health Questionnaire (GHQ-28)). All the evaluations were recorded at baseline, at 4 weeks, and at 20 weeks. According to this study, PD patients’ QoL and perceived psychological well-being improved after early spa interventions. On the SF-36, spa therapy showed a significant effect both on the physical and on the mental dimension (*P* = 0.03 and *P* = 0.001, respectively). All dimensions of the PDQ-39, excepts social support, tended to be improved in the spa period at the 4-week follow-up. After 4 weeks, the mean change in Part IV of UPDRS in the spa period was significantly different from those in the non-spa period; however, this significance was not maintained after 20 weeks. The mean change in the GHQ-28 global score was substantially improved in the spa phase, with the two subscores anxiety and insomnia and severe depression considerably higher after 4 weeks (*P* = 0.006 and *P* = 0.04, respectively). However, also these effects were not maintained after 20 weeks.

Brefel-Courbon’s study was the only one found to consider cost-effectiveness. The spa intervention was shown to decrease health-related expenditure, although with a small reduction in direct medical costs. Even if the spa treatment had a cost, the cost of hospitalization and paramedical care was lower than during the non-spa period (Brefel-Courbon et al. 2003).

Masiero et al. considered, in a retrospective study, PD patients at stages 1, 2, or 3 of the Hoehn and Yahr Scale, admitted to a thermal rehabilitation center in Abano Terme (Veneto region, Italy). The patients underwent an aquatic thermal training conducted in a small group of 3 to 5 participants, with 45-min sessions, twice a week on non-consecutive days, over 4 weeks. During the training, the patients were allowed to continue their usual motor activities and medication. The rehabilitative interventions consisted of functional re-education, kinesitherapy in a salso-bromo-iodic thermal pool, diet, health education, and cognitive behavioral advice. The evaluation protocols were assessed the day of admission and at the end of the training in OFF state. The Berg Balance Scale (BBS) and the Tinetti balance assessment tool Scale were defined as the primary outcomes. Enrolled subjects obtained statistically significant improvement in the BBS and in the Tinetti balance assessment tool Scale. Moreover, the UPDRS, the Parkinson’s Disease Questionaire-8, and the Balance Evaluation Systems Test resulted improved after the treatment (Masiero et al. 2019).

In the Euganean basin of Abano and Montegrotto (Veneto region, Italy), recently another clinical trial was conducted to assess cognitive and motor health, functional capacities, and QoL among PD patients before and after an intense rehabilitation program in thermal water (12 45-min sessions, twice a week for 6 weeks). Participants, characterized by a Hoehn and Yahr stage 2 or 3 in OFF-state, were arranged in groups of five, with two physical therapists supervising each session. Thermal aquatic program was focused on improving balance, posture, and gait. The Mini Balance Evaluation System Test and the PDQ-39 were regarded as the primary outcomes. Secondary evaluation measures assessed motor symptoms, QoL, and psychological well-being. Following therapy, participants maintained high cognitive and functional condition. The overall balance of all subjects improved considerably (*p* = 0.01). The PDQ-39 improved considerably after rehabilitation (*p* = 0.038), with the significance driven by features highly related to motor status (Di Marco et al. 2022).

In all the studies considered, no adverse events were recorded (Brefel-Courbon et al. 2003; Di Marco et al. 2022; Masiero et al. 2019).

Table 2 summarizes the results of the different studies in more detail.

## Discussion

Despite the heterogeneity in the treatment modalities reported, the small number of trials, and the little number of patients considered represents a potential limitation of the conclusion on the real evidence-based efficacy of the spa environment for neurodegenerative patients, initial evidence seems to show a promising role of rehabilitative interventions conducted in the thermal environment for patients affected by neurodegenerative disorders and, in particular, by PD.

The studies found were homogeneous in terms of the condition addressed, namely PD, and the stage of disease progression. In particular, the studies included in the review seem to suggest that the spa environment can represent a safe environment for conducting rehabilitation programs in patients with PD at the early stages according to the Hoehn and Yahr classification.

There is a substantial lack of standardization in the spa rehabilitative protocols documented in literature. This finding makes it difficult to compare the studies and determine if one regimen surpasses the other in terms of clinical benefit. However, the primary aim of this scoping review was to highlight the available literature on the topic, opening the path for future research that may address appropriate applications of spa rehabilitative interventions for neurodegenerative diseases.

Until date, PD care has been mostly focused on pharmacological therapy; however, therapeutic exercise has recently been emphasized as an adjunct to pharmacological treatment. Previous research on the relationship between PD and exercise has revealed that physical activity can contribute to delay the onset of the disease and slow its progression. In addition, exercise helps to improve motor impairment and balance, as well as cognitive decline and QoL. Furthermore, it has recently been hypothesized that exercise may selectively rebalance basal ganglia function, allowing levodopa to partially restore dopaminergic tonus (Xu et al. 2019). The preliminary results obtained in the thermal setting seem to reconfirm the benefits obtained with exercise in non-thermal water. So far, these preliminary findings appear to show that rehabilitative intervention in the spa setting have the potential to alleviate motor symptoms such as rigidity, postural instability, and balance impairments, as well as improve the QoL of these patients (Clerici et al. 2019; Vivas et al. 2011; Volpe et al. 2014, 2017). Exercise in thermal water, in fact, combines the particular chemical effects of mineral-rich water with the physical consequences of immersion. Just as in non-thermal water, exercise in thermal water can help PD patients regain lower limb strength and motor function by promoting more extensive movements with less muscular effort, reducing joint compression and weight load (Fioravanti et al. 2011). These effects could act positively on bradykinesia, one of the cardinal symptoms of PD, helping to improve patients’ autonomy and QoL. Furthermore, thanks to the buoyancy force and the hydrostatic pressure fear of falling can be reduced during water-based exercises (Masiero et al. 2018; Vivas et al. 2011). In the study conducted by Di Marco et al., no decline in activities of daily living was observed at the end of treatment, suggesting the possibility that exercise in the spa environment may help delay the worsening of disabilities (Di Marco et al. 2022). In addition, the spa setting, being suitable to provide sensory stimuli and external triggering, could offer a further contribution at the early stage of the disease, helping to change the movement from automatic to voluntarily controlled through the modulation of visuo-cerebellar and reticulo-spinal alternative motor pathways (Dacre et al. 2021). Water temperature, on the other hand, can help in the reduction of muscle rigidity due to its effects on the autonomous nervous system and, in particular, on the relaxation that occurs as a result of the parasympathetic system activation (Becker et al. 2009; Mur Gimeno et al. 2020).

The results on QoL confirm the findings of previous research. Numerous studies in the musculoskeletal field have so far shown that spa treatments can have a positive effect on QoL (Cheleschi et al. 2022; Devereux et al. 2005; Maccarone et al. 2020); however, few data specifically address the improvement of QoL in neurological patients after aquatic or spa therapy. In a group of patients with acquired brain damage who underwent non-thermal aquatic Ai Chi therapy, statistically significant differences were detected on QoL at the end of the treatment (Pérez-de la Cruz 2020). In subjects attending spa facilities the natural, relaxing environment seems to play an additional role in improving QoL, mood, and anxiety. The rationale for these changes is that balneotherapy can contribute in reducing stress and lowering neuronal excitation, as well as alterations in hormones such as cortisol and endogenous opiates (Pérez-de la Cruz 2020; Yang et al. 2018). Furthermore, the spa environment can work therapeutically on psychological issues, having a positive action on resilience and depression mediated by an increase in adrenocorticotropic hormone, cortisol, prolactin, growth hormone, and endorphin levels following balneotherapy (Antonelli & Donelli 2018; Fioravanti et al. 2017; Gálvez et al. 2018; Masiero & Maccarone 2021). In addition, traditional spa setting can restrict the social isolation often experienced by neurodegenerative patients and encourage social integration between healthy subjects and people with disabilities (Maccarone et al. 2021a, b; Masiero & Maccarone 2021; Secher et al. 2009). Furthermore, balneotherapy can represent also a potential moment of respite for the caregiver (Secher et al. 2009).

Finally, first data on the topic seem to suggest that rehabilitative interventions in the spa setting for PD patients are cost-effective, allowing the costs associated with rehabilitation to be reduced, even if slightly (Brefel-Courbon et al. 2003). These findings need to be further investigated because, if applied on a large scale, it could help reduce a major economic burden (Brefel-Courbon et al. 2003).

Unfortunately, no studies analyzing rehabilitation treatments in the spa environment for patients with other neurodegenerative diseases except PD have been found. Aquatic interventions have been demonstrated to be practicable, safe, and associated with significant improvement in motor function and spasticity in patients with multiple sclerosis (Plecash & Leavitt 2014; de Sa et al. 2011). However, spa rehabilitative interventions in multiple sclerosis are still discussed in clinical practice, as the effect of warm water temperature on the worsening of symptoms is unclear (Christogianni et al. 2018). Future pre-clinical and clinical studies should investigate these aspects in order to implement safe and beneficial rehabilitation protocols in neurodegenerative patients.

Overall, this scoping review led to highlight a substantial lack of studies evaluating rehabilitative treatments in the thermal environment for patients with neurodegenerative diseases, even if applications in clinical practice are increasing (Masiero et al. 2020a, b). However, the data presented in this review appear to be the first to indicate the efficacy of treatments performed in the thermal environment for neurodegenerative patients. As a result, our findings are significant because, while encouraging, they indicate the need for more research in this area.

## Conclusion

Although due to the small number and the heterogeneity of studies it is not yet possible to define a univocal protocol for the spa rehabilitative treatment of neurodegenerative patients, initial evidence seems to show a relevant role of thermal water exercise in the prevention and delay of disabilities at the early stage of neurodegenerative diseases. In particular, thermal aquatic rehabilitation might become a useful strategy in the rehabilitation and disabilities prevention program of PD patients providing improvements in motor function, balance, QoL, and perception of psychological well-being. In addition, rehabilitation in the spa environment seems cost-effective. Therefore, thermal water rehabilitation should represent a proposal to be further developed for this type of patients on the basis of further clinical studies. Research in the spa setting for additional neurodegenerative disorders is also required in order to establish appropriate preventive-rehabilitative programs.
